# Risk of involuntary admission among first-generation ethnic minority groups with early psychosis: a retrospective cohort study using health administrative data

**DOI:** 10.1017/S2045796019000556

**Published:** 2019-10-15

**Authors:** Rebecca Rodrigues, Arlene G. MacDougall, Guangyong Zou, Michael Lebenbaum, Paul Kurdyak, Lihua Li, Salimah Z. Shariff, Kelly K. Anderson

**Affiliations:** 1 Department of Epidemiology & Biostatistics, Schulich School of Medicine & Dentistry, The University of Western Ontario, London, ON, Canada; 2 Department of Psychiatry, Schulich School of Medicine & Dentistry, The University of Western Ontario, London, ON, Canada; 3 Robarts Research Institute, Schulich School of Medicine & Dentistry, The University of Western Ontario, London, ON, Canada; 4 ICES, Toronto, ON, Canada; 5 Department of Psychiatry, Faculty of Medicine and Institute for Health Policy, Management and Evaluation, University of Toronto, Toronto, ON, Canada; 6 Centre for Addiction and Mental Health, Toronto, ON, Canada

**Keywords:** Ethnicity, first-episode psychosis, health administrative data, immigrant, involuntary hospitalisation, mental health services, refugee

## Abstract

**Aims:**

Ethnic minority groups often have more complex and aversive pathways to mental health care. However, large population-based studies are lacking, particularly regarding involuntary hospitalisation. We sought to examine the risk of involuntary admission among first-generation ethnic minority groups with early psychosis in Ontario, Canada.

**Methods:**

Using health administrative data, we constructed a retrospective cohort (2009–2013) of people with first-onset non-affective psychotic disorder aged 16–35 years. This cohort was linked to immigration data to ascertain migrant status and country of birth. We identified the first involuntary admission within 2 years and compared the risk of involuntary admission for first-generation migrant groups to the general population. To control for the role of migrant status, we restricted the sample to first-generation migrants and examined differences by country of birth, comparing risk of involuntary admission among ethnic minority groups to a European reference. We further explored the role of migrant class by adjusting for immigrant vs refugee status within the migrant cohort. We also explored effect modification of migrant class by ethnic minority group.

**Results:**

We identified 15 844 incident cases of psychotic disorder, of whom 19% (*n* = 3049) were first-generation migrants. Risk of involuntary admission was higher than the general population in five of seven ethnic minority groups. African and Caribbean migrants had the highest risk of involuntary admission (African: risk ratio (RR) = 1.52, 95% CI = 1.34–1.73; Caribbean: RR = 1.58, 95% CI = 1.37–1.82), and were the only groups where the elevated risk persisted when compared to the European reference group within the migrant cohort (African: RR = 1.24, 95% CI = 1.04–1.48; Caribbean: RR = 1.29, 95% CI = 1.07–1.56). Refugee status was independently associated with involuntary admission (RR = 1.16, 95% CI = 1.02–1.32); however, this risk varied by ethnic minority group, with Caribbean refugees having an elevated risk of involuntary admission compared with Caribbean immigrants (RR = 1.72, 95% CI = 1.15–2.58).

**Conclusions:**

Our findings are consistent with the international literature showing increased rates of involuntary admission among some ethnic minority groups with early psychosis. Interventions aimed at improving pathways to care could be targeted at these groups to reduce disparities.

## Introduction

International evidence has demonstrated notably higher rates of involuntary admission among some ethnic minority groups, with the largest risk observed among Black-Caribbean and Black-African patients compared with White patients, and moderate risk for South Asian patients (Bhui *et al*., 2003; Singh *et al*., 2007; Halvorsrud *et al*., 2018; Barnett *et al*., 2019). Early studies on ethnic differences in involuntary admission in the context of first-episode psychosis did not tend to find these differences (Cole *et al*., 1995; Burnett *et al*., 1999; Goater *et al*., 1999), leading to the theory that this excess develops over time as a result of repeated negative interactions with mental health services (Burnett *et al*., 1999; Singh *et al*., 2007). Subsequent larger-scale studies have provided evidence that disparities among Black-Caribbean and Black-African patients are evident at the first episode (Morgan *et al*., 2005; Mann *et al*., 2014*b*) – findings supported by recent meta-analytic evidence showing no subgroup differences between the risk of first involuntary admission *v*. readmission (Halvorsrud *et al*., 2018). Less evidence exists regarding involuntary admission among other ethnic minority groups with early psychosis, given that these groups tend to be underrepresented in prior studies (Anderson *et al*., 2014; Mann *et al*., 2014*a*).

These disparities in service experiences among ethnic minority groups early in the course of psychotic illness may have long-term consequences. People with an involuntary admission are more likely to experience control interventions within the first 3 days of hospitalisation and tend to have a shorter length of stay (Rodrigues *et al*., 2019). It has been suggested that these initial negative interactions may initiate a ‘vicious cycle of negative experiences, coercion, disengagement, relapse and so on’ (Morgan *et al*., 2004). Indeed, experiencing an involuntary admission is a strong predictor of readmissions occurring on an involuntary basis (Kallert *et al*., 2008). Furthermore, 5- and 10-year follow-up of Black-African and Black-Caribbean groups have shown a course of illness characterised by persistent negative service contacts, including more frequent hospital admissions, involuntary admissions, police involvement, and longer periods of admission compared with White patients (Ajnakina *et al*., 2017; Morgan *et al*., 2017).

Despite more than 20 years of research on inequities in involuntary admission among ethnic minority groups, the vast majority of prior research focused on early psychosis has been from the UK and there remains a dearth of international evidence. Among the limited studies, findings are often not consistent with the trends observed in the UK (Selten and Sijben, 1994; Turner *et al*., 2006; Archie *et al*., 2010). Additionally, many prior studies have been done in the context of early psychosis intervention services and are likely not representative of the broader early psychosis population (Anderson *et al*., 2018).

It is also unclear to what extent migrant status impacts the risk of involuntary admission in early psychosis. Meta-analytic evidence of both psychotic and non-psychotic involuntary admissions indicates that migrant groups are significantly more likely to be involuntarily admitted compared with non-migrant groups (Barnett *et al*., 2019). It is plausible that ethnicity and migrant status may have differential effects on the risk of involuntary admission. Migrant status may be related to issues with navigating the health care system due to lack of knowledge, economic, or language barriers (Thomson *et al*., 2015) which may increase the likelihood of involuntary admission. The effect of ethnicity, however, may be related to cultural barriers, such as differences in the perceptions of mental illness, appropriateness of services, or racial discrimination (Thomson *et al*., 2015). Furthermore, no studies examining involuntary admission in early psychosis have examined refugees within ethnic minority groups. Refugees are more likely than non-refugee migrants to face adversity with respect to pre- and post-migratory exposures, and are at higher risk of a variety of psychiatric disorders compared with the general population, including psychotic disorders (Kirmayer *et al*., 2011; Anderson *et al*., 2015*a*). Understanding differences in mental health service experiences between migrants and ethnic minority groups, and within migrant classes, has important implications for mental health service provision and policy.

The primary aim of this study was to use large-scale health administrative databases to examine the risk of involuntary hospitalisation among first-generation ethnic minority groups in young people with early psychosis in Ontario (Canada). The secondary aim was to examine the risk of involuntary admission within a subsample restricted to first-generation migrants, in order to isolate the effects of ethnicity and to examine the role of refugee status on the risk of involuntary admission.

## Methods

### Data sources

We used health administrative data linked at the patient level and housed at ICES – an independent, non-profit research institute whose legal status under Ontario's health information privacy law allows it to collect and analyse health care and demographic data, without consent, for health system evaluation and improvement. The Ontario Mental Health Reporting System (OMHRS) includes data on all admissions to designated adult inpatient psychiatric beds and the Discharge Abstract Database (DAD) contains data on all hospitalisations to non-psychiatric beds. The Ontario Health Insurance Plan (OHIP) Claims Database includes billing claims for physician services and outpatient visits. The National Ambulatory Care Reporting System (NACRS) includes information on emergency department (ED) visits. The Registered Persons Database (RPDB) contains demographic information on all people eligible for OHIP. Data on first-generation migrants (people born outside of Canada) were obtained from the Immigration, Refugees, and Citizenship Canada (IRCC) Permanent Resident database. These datasets were linked using unique encoded identifiers and analysed at ICES.

### Study design and case definition

We constructed a retrospective cohort of incident cases of non-affective psychotic disorder aged 16–35 years in Ontario over a 5-year period (2009–2013, inclusive) using a validated algorithm: (i) one hospitalisation in OMHRS or DAD with a primary discharge diagnosis of schizophrenia, schizoaffective disorder, or psychosis not otherwise specified (NOS); or (ii) at least two physician billings or ED visits for schizophrenia, schizoaffective disorder, or psychosis NOS in a 12-month period (Kurdyak *et al*., 2015). The first billing record identified was used to obtain the index diagnosis and the index date (i.e. the first date of contact with services for non-affective psychosis). We excluded prevalent cases, identified by contact with services with a diagnostic code for non-affective psychosis within the 20-year period prior to 2009. We excluded cases *post hoc* where the diagnosis at first hospitalisation changed to affective or organic psychosis, as well as migrants from Oceania or the USA due to small numbers (*n*  =  67). We restricted our analyses to people living in urban settings (population of 10 000 or more), given that 99% of first-generation migrant groups in our sample resided in urban areas, and we have previously shown urban-rural variations in service use for early psychosis (Anderson and Kurdyak, 2017). A complete list of the codes used to define the study cohort and variables is available in the online Supplementary Table S1. We followed the RECORD guidelines for observational studies using administrative data (online Supplementary Table S2; Benchimol *et al*., 2015).

### Classification of ethnicity and refugee status

We classified ethnic minority groups based on country of birth from IRCC Permanent Resident database. We used classifications for the country of birth developed by Statistics Canada (Statistics Canada, 2011) and further amalgamated these groupings for consistency with prior studies (Anderson *et al*., 2017). The amalgamated classifications included the following groupings: (i) European, including people from Northern (e.g. England, Scotland), Southern (e.g. Italy, Greece), Western (e.g. France, Germany) and Eastern (e.g. Poland) European countries, and Russia; (ii) Caribbean (e.g. Jamaica, Cuba, Haiti, Puerto Rico, and Bermuda); (iii) South Asian (e.g. India, Pakistan, Bangladesh, Nepal, Sri Lanka); (iv) East Asian (e.g. China, Japan, Philippines, Korea, Thailand, Taiwan, Vietnam); (v) Latin American, including people from Central and South America (e.g. Mexico, Argentina, Brazil); (iv) North African and Middle East (e.g. Egypt, Morocco, Turkey, Israel, Syria, Afghanistan); and (vi) African, including people from sub-Saharan Africa (e.g. Kenya, Ethiopia, Ghana, Nigeria, Zimbabwe). All remaining people in our cohort were classified as the general population reference group, which consisted of a heterogeneous group of non-migrants, second-generation or higher migrants and long-term residents. We differentiated between immigrants and refugees in IRCC records based on the immigration class to which they applied.

### Ascertainment of outcome

We followed cases over the 2-year period subsequent to the index date to identify the first psychiatric hospitalisation for any mental health reason. In cases where the index diagnosis occurred by hospitalisation, this event was used as the first hospitalisation. We identified involuntary admissions, defined as a patient admitted under a Form 1 (Application for Psychiatric Assessment) or a Form 3 (Certificate of Involuntary Admission) under the Ontario *Mental Health Act* (Ontario Hospital Association, 2016). Both forms permit detention of the patient within hospital, with a Form 1 being valid for up to 72 h and a Form 3 being valid for up to 2 weeks (Ontario Hospital Association, 2016). Cases having an involuntary admission at the first psychiatric hospitalisation were defined as having the outcome, whereas cases never admitted or admitted under voluntary, forensic, or informal criteria were in the comparison group. We have previously shown that the majority of hospitalisations in our cohort are on an involuntary basis (approximately 81%; Rodrigues *et al*., 2019), therefore we did not examine hospitalisation as a separate outcome in our analysis.

### Sociodemographic and clinical variables

We obtained data on sex and age at the index date from RPDB. We obtained forward sortation areas from RPDB to derive average neighbourhood-level income adjusted for household size and housing costs, which was determined using census data and divided into quintiles. We identified the index diagnosis (schizophrenia spectrum disorder or psychosis NOS) from the health administrative records.

### Data analysis

We calculated standardised differences to compare characteristics between groups, with a difference of >0.1 suggestive of significant between-group differences (Austin, 2009). For all analyses, we used modified Poisson regression models to estimate risk ratios (RRs) and associated 95% confidence intervals (CIs; Zou, 2004). We estimated unadjusted RRs, and RRs adjusted for age, sex, neighbourhood-level income quintile and index diagnosis. We considered 95% CIs excluding unity to be statistically significant. Patients with missing data were excluded from our regression analyses (<1% of the sample). All analyses were conducted in SAS Enterprise Guide (Version 6.1, SAS Institute Inc., Cary, NC, USA).

For our primary analysis, we estimated RRs for the risk of involuntary admission in ethnic minority groups compared with the general population. For our secondary analyses, we limited the cohort to first-generation migrants to control for migrant status, which was not possible within the full cohort due to collinearity with ethnic minority group. Within this cohort, we estimated RRs for the risk of involuntary admission among first-generation ethnic minority groups compared with the European migrant group to assess whether there were differences in risk among first-generation ethnic minority groups. In our multivariable model, we further adjusted for migrant class (immigrant *v*. refugee) to determine whether refugees had an elevated risk of involuntary admission. To assess the presence of effect modification of migrant class by ethnic minority group, we added an interaction term for ethnic minority group × migrant class to the model. We conducted a sensitivity analysis to determine whether there were similar ethnic differences in all hospitalisations, as opposed to only involuntary admissions.

## Results

After exclusions, we identified 15 844 incident cases of non-affective psychosis over a 5-year period (online Supplementary Figure S1). The cohort characteristics are summarised in Table 1. First-generation migrants accounted for 19% of the cohort (*N*  =  3049). Migrant groups were older at first diagnosis compared with the general population. The majority of cases were male in most groups; however, South and East Asian migrants had a higher proportion of females compared with the general population. All migrant groups had a higher proportion of people residing in the lowest two income quintiles compared with the general population, with the Caribbean and African groups having the highest proportions. Migrant groups from Africa and from North Africa and the Middle East had the largest proportion of refugees, with 50% and 43%, respectively.

**Table 1.** Characteristics of the early psychosis cohort by first-generation ethnic minority group

	General population *N* = 12 795	European *N* = 547	African *N* = 405	Caribbean *N* = 303	South Asian *N* = 652	East Asian *N* = 450	Latin American *N* = 227	North African and Middle East *N* = 465
	*N* (%)	*N* (%)	*N* (%)	*N* (%)	*N* (%)	*N* (%)	*N* (%)	*N* (%)
Age, mean (s.d.)	24.0 (5.6)	25.2 (5.1)^a^	25.1 (5.0)^a^	25.0 (4.9)^a^	26.4 (5.5)^a^	25.7 (5.4)^a^	25.3 (5.4)^a^	25.3 (5.2)^a^
Sex								
Female	4508 (35)	201 (37)	136 (34)	103 (34)	287 (44)^a^	208 (46)^a^	86 (38)	142 (31)
Male	8287 (65)	346 (63)	269 (66)	200 (66)	365 (56)^a^	242 (54)^a^	141 (69)	323 (69)
Income quintile								
5 (highest)	2048 (16)	72 (13)	18 (4)^a^	13 (4)^a^	35 (5)^a^	41 (9)^a^	19 (8)^a^	45 (10)^a^
4	2294 (18)	100 (18)	39 (10)^a^	28 (9)^a^	82 (13)^a^	66 (15)	26 (12)^a^	75 (16)
3	2360 (19)	113 (21)	49 (12)^a^	40 (13)^a^	133 (21)	79 (18)	48 (21)	77 (17)
2	2690 (21)	106 (19)	95 (24)	74 (25)	167 (26)^a^	130 (29)^a^	45 (20)	93 (20)
1 (lowest)	3344 (26)	155 (28)	203 (50)^a^	147 (49)^a^	231 (36)^a^	133 (30)	88 (39)^a^	172 (37)^a^
Refugee status	N/A	99 (18)	202 (50)	18 (6)	110 (17)	52 (12)	60 (26)	202 (43)
Index diagnosis								
Schizophrenia spectrum	6254 (49)	243 (44)^a^	203 (50)	137 (45)	360 (55)^a^	224 (50)	129 (57)^a^	252 (54)^a^
Psychosis NOS	6541 (51)	304 (56)^a^	202 (50)	166 (55)	292 (45)^a^	226 (50)	98 (43)^a^	213 (46)^a^

s.d., standard deviation; NOS, not otherwise specified.

a Standardised difference >0.10 compared with the general population.

### Risk of involuntary admission: full cohort

Within the 2-year follow-up, 35% of the cohort (*N*  =  5610) had a first psychiatric hospitalisation, with a median time from diagnosis to hospitalisation of 43 days (interquartile range  =  1–263 days). Within the full cohort, 26% of people (*N*  =  4131) experienced an involuntary first admission. In both unadjusted and adjusted models, European, African, Caribbean and East Asian migrants had a higher risk of involuntary admission compared with the general population (Table 2). Migrants from North Africa and Middle East had significantly elevated risk of involuntary admission compared with the general population only in the adjusted model (Table 2). The risk of involuntary admission was highest among migrants from the Caribbean (RR  =  1.58, 95% CI = 1.37–1.82) and Africa (RR  =  1.52, 95% CI = 1.34–1.73; Table 2). For our sensitivity analysis of ethnic differences in any hospitalisation, we largely observed trends consistent with the involuntary admission outcome with smaller effect sizes (online Supplementary Table S3).

**Table 2.** Risk of involuntary admission among first-generation ethnic minority groups within the early psychosis cohort

Ethnic minority group	*N*	Involuntary *N* (%)	Unadjusted RR	95% CI	Adjusted RR^a^	95% CI
General population	12 795	3198 (25)	Reference	–	Reference	–
European	547	164 (30)	1.20	1.05–1.37	1.22	1.07–1.39
African	405	151 (37)	1.49	1.31–1.70	1.52	1.34–1.73
Caribbean	303	119 (39)	1.57	1.36–1.81	1.58	1.37–1.82
South Asian	652	168 (26)	1.03	0.90–1.18	1.14	1.00–1.32
East Asian	450	141 (31)	1.25	1.09–1.44	1.32	1.15–1.52
Latin American	227	57 (25)	1.00	0.80–1.26	1.08	0.86–1.35
North African and Middle East	465	133 (29)	1.14	0.99–1.33	1.21	1.04–1.40

RR, risk ratio; CI, confidence interval.

a Adjusted for age, gender, income quintile and diagnosis (schizophrenia spectrum disorder *v*. psychosis NOS).

### Risk of involuntary admission: migrant cohort

Within the migrant cohort, the risk of involuntary admission remained elevated in African and Caribbean ethnic minority groups relative to European migrants (African: RR  =  1.24, 95% CI = 1.04–1.48; Caribbean: RR  =  1.29, 95% CI = 1.07–1.56; Table 3). However, South Asian, East Asian and North African and Middle Eastern groups no longer had significantly higher risk of involuntary admission when compared with the European group (Table 3). After accounting for migrant class in the model, only the Caribbean group had a higher risk of involuntary admission compared with the European group (RR  =  1.32, 95% CI = 1.09–1.60). Refugee status was associated with a 16% increased risk in involuntary admission (RR  =  1.16, 95% CI = 1.02–1.32; Table 3).

**Table 3.** Risk of involuntary admission among first-generation ethnic minority groups within the migrant cohort, compared with a European reference group

			Unadjusted		Adjusted model 1^a^		Adjusted model 2^b^	
Ethnic minority group	*N*	Involuntary *N* (%)	RR	95% CI	RR	95% CI	RR	95% CI
European	547	164 (30)	Reference	–	Reference	–	Reference	–
African	405	151 (37)	1.24	1.04–1.49	1.24	1.04–1.48	1.19	0.99–1.43
Caribbean	303	119 (39)	1.31	1.08–1.58	1.29	1.07–1.56	1.32	1.09–1.60
South Asian	652	168 (26)	0.86	0.72–1.03	0.91	0.76–1.09	0.91	0.76–1.09
East Asian	450	141 (31)	1.05	0.87–1.26	1.07	0.89–1.29	1.08	0.90–1.30
Latin American	227	57 (25)	0.84	0.65–1.08	0.87	0.67–1.12	0.86	0.67–1.11
North African and Middle East	465	133 (29)	0.95	0.79–1.16	0.98	0.81–1.19	0.94	0.78–1.15
Migrant status								
Immigrant	2306	679 (29)	–	–	–	–	Reference	
Refugee	743	254 (34)	–	–	–	–	1.16	1.02–1.32

RR, risk ratio; CI, confidence interval.

a Adjusted for age, sex, income quintile and diagnosis (schizophrenia spectrum disorder *v*. psychosis NOS).

b Migrant status added to the adjusted model 1.

Findings examining effect modification of migrant status by ethnic minority group are summarised in Table 4. Within the group of migrants from the Caribbean, refugee status was associated with a 72% increased risk of involuntary first admission compared with Caribbean immigrants (RR  =  1.72, 95% CI = 1.15–2.58). There was also some evidence that refugees from North Africa and Middle East had an elevated risk of involuntary admission compared with immigrants from this region (RR  =  1.30, 95% CI  =  0.98–1.73).

**Table 4.** Modification of the effect of migrant status on the risk of involuntary admission by ethnic minority group within the migrant cohort

Ethnic minority group	Immigrants		Refugees		Refugee status within each ethnic minority group
	*N* involuntary/no involuntary admission	Adjusted RR (95% CI)	*N* involuntary/no involuntary admission	Adjusted RR (95% CI)	Adjusted RR (95% CI)
European	130/318	Reference	29/70	Reference	1.03 (0.75–1.43)
African	69/134	1.24 (0.99–1.55)	72/130	1.24 (0.88–1.74)	1.02 (0.80–1.31)
Caribbean	104/181	1.25 (1.02–1.54)	11/7	2.10 (1.30–3.40)	1.72 (1.15–2.58)
South Asian	131/411	0.90 (0.73–1.10)	31/79	0.98 (0.64–1.48)	1.11 (0.80–1.55)
East Asian	116/282	1.05 (0.85–1.29)	20/32	1.26 (0.80–1.98)	1.23 (0.85–1.79)
Latin American	36/131	0.82 (0.61–1.12)	18/42	1.00 (0.62–1.62)	1.25 (0.78–2.00)
North African and Middle East	65/198	0.87 (0.68–1.12)	60/142	1.10 (0.78–1.57)	1.30 (0.98–1.73)

RRs are adjusted for age, sex, income quintile and diagnosis (schizophrenia spectrum disorder *v*. psychosis NOS) and with interaction term in the model (ethnic minority group × migrant status).

## Discussion

Among young people with early psychosis in Ontario, most first-generation ethnic minority groups had a higher risk of a first involuntary admission within 2 years of diagnosis compared with the general population. African and Caribbean groups were the most likely to experience an involuntary admission, with a 52% and 58% increased risk of involuntary admission, respectively. In our analyses restricted to first-generation migrants, the risk of involuntary admission remained significantly elevated in African and Caribbean migrants compared with European migrants, however, effect sizes were attenuated relative to the general population comparison group. Refugee status was associated with an increased risk of involuntary admission, however, this elevated risk varied by ethnic minority group, with Caribbean refugees, in particular, having a higher risk compared with immigrants from the same region.

Our findings implicate ethnic differences in the risk of involuntary admission in early psychosis. However, our comparison of first-generation ethnic minority groups with a heterogeneous general population group, which includes long-term residents and second-generation or higher migrants, likely underestimates the effects of ethnicity. Importantly, our findings do not fully account for mental health care experiences across the full spectrum of ethnic minorities. Despite this limitation, our findings closely resemble trends observed in the UK from studies that used self-reported ethnicity and were not limited to first-generation migrants. In particular, studies from the UK have similarly observed that Caribbean and African groups have a higher risk of involuntary admission compared with a White reference group – an inequity that exists early in the course of illness (Morgan *et al*., 2005; Mann *et al*., 2014*b*; Halvorsrud *et al*., 2018). Also consistent with UK evidence is our finding that South Asian groups do not have elevated risk of involuntary first admission (Cole *et al*., 1995; Burnett *et al*., 1999; Goater *et al*., 1999; Mann *et al*., 2014*b*). Meta-analytic evidence from the UK indicates that South Asian groups have an elevated risk of readmission, but not first admission (Halvorsrud *et al*., 2018). Our finding that most ethnic minority groups are more likely to have involuntary first admission conflicts with previous Canadian evidence (Archie *et al*., 2010). This discrepancy may be related to the relatively small sample size of the previous study, as other larger studies in Ontario, not restricted to early psychosis, have also observed ethnic differences in involuntary admission (Chiu *et al*., 2016; Rotenberg *et al*., 2017). Beyond these trends, our findings have limited comparability to previous studies due to differences in ethnic minority groupings. To our knowledge, our study is the first to examine the risk of involuntary admission among European, East Asian, North African and Middle Eastern and Latin American groups. The considerable variation in the risk of involuntary admission among different ethnic minority groups not previously examined in the early psychosis literature highlights the importance of focused research into the different needs and experiences of well-defined ethnic minority groups, as previously described by Mann *et al*. (2014*b*).

When we limited the cohort to first-generation migrants and used a European-migrant reference group, the risk of involuntary admission remained elevated only for African and Caribbean migrants; however, the effect size was attenuated as compared with the general population reference group. This suggests that mechanisms related to the elevated risk of involuntary admission for ethnic minority groups may be partially explained by migrant status, rather than ethnic differences *per se*. Migratory factors may be particularly relevant for South Asian, East Asian and North African and Middle Eastern groups, which no longer had a significantly higher risk of involuntary admission when compared with European migrants. However, the persistent elevated risk for African and Caribbean groups as compared with Europeans may suggest some influence of ethnicity-related factors, or it may be that common migratory and resettlement related-effects are more severe among African and Caribbean groups compared with Europeans.

We observed that refugee status was associated with a higher risk of involuntary admission, which we have also shown previously (Rodrigues *et al*., 2019). However, given the relatively large proportion of African refugees in our cohort, it may be that this finding is partially driven by elevated risk among all African migrants, rather than refugee status among migrants from any one region. Indeed, results from our analysis of effect modification suggest the importance of refugee status varies by region of birth. For African migrants, there was no difference in the risk of involuntary admission among African refugees compared with African immigrants, suggesting that all African migrants have elevated the risk of involuntary admission. However, refugee status in Caribbean migrants was associated with a large increase in the risk of involuntary admission compared with Caribbean immigrants. We also observed an elevated risk among North African and Middle Eastern refugees, albeit the confidence interval includes the possibility of a null effect. Due to sample size limitations, our interaction analysis may have been underpowered to detect statistically significant differences. Further studies are needed to investigate the role of refugee status among migrants from different regions, particularly in Caribbean groups, which was the smallest group of refugees in our cohort (*n*  =  18).

### Explaining the differences

Many theories as to why ethnic minority groups are at higher risk of involuntary admission in early psychosis have been previously discussed, mostly pointing to the role of upstream factors occurring prior to admission (Harrison *et al*., 1989; Cole *et al*., 1995; Goater *et al*., 1999; Morgan *et al*., 2005; Mann *et al*., 2014*a*, 2014*b*). However, there is limited evidence to support these hypotheses. Pathways to care have been implicated, given that Black ethnic minority groups are less likely to have pathways with general practitioner (GP) involvement, and more likely to have police involvement (Anderson *et al*., 2014). However, evidence regarding whether such differences explain the excess risk of involuntary admission in ethnic minority groups has been inconsistent (Burnett *et al*., 1999; Morgan *et al*., 2005; Mann *et al*., 2014*b*). Other theories include social isolation (i.e. the absence of someone to facilitate help-seeking; Harrison *et al*., 1989; Cole *et al*., 1995; Burnett *et al*., 1999; Morgan *et al*., 2005), more severe clinical presentation (Goater *et al*., 1999; Morgan *et al*., 2005) and racial discrimination in which Caribbean patients are more likely to be perceived as violent or threatening (Harrison *et al*., 1989; Koffman *et al*., 1997; Goater *et al*., 1999; Morgan *et al*., 2005). Only one study to date has examined all of these factors in their analysis and findings suggest these differences do not explain the excess risk of involuntary admission observed among ethnic minority groups in early psychosis (Morgan *et al*., 2005). More evidence exists from studies broadly examining all involuntary admissions, and direct evidence to date lends the most support to similar explanations, such as increased police contact, absence of or mistrust of GPs, increased perceived risk of violence and ethnic disadvantages (Barnett *et al*., 2019). Further investigation may provide evidence to support these theories in the context of involuntary admission in early psychosis.

Taken together, it is likely that the mechanisms underlying our findings are complex, and the potential causal mechanisms are likely interrelated through different pathways, which may have differential effects for different ethnic minority groups. Future studies aimed at understanding the mechanisms behind the excess risk of involuntary admission among ethnic minority groups with early psychosis are warranted in order to inform strategies to reduce these inequities. Qualitative research may be particularly informative, as noted by Bhui and colleagues, who call for experts to ‘gather lived experiences and hear hidden voices, which we argue hold clues for how health inequalities arise and are sustained, how racism operates and how we can empower people and communities to make best use of the cultural affordances and community assets at their disposal’ (Bhui *et al*., 2018).

### Limitations

The use of health administrative data only allowed us to examine cases of non-affective psychosis who were in contact with the health system, therefore our study is not a true population-based assessment. We used a case definition algorithm with high sensitivity (Kurdyak *et al*., 2015), therefore there is a risk of false positives within our cohort. As well, prevalent cases where the first diagnosis of psychosis occurred outside of Ontario would be misclassified as incident cases in our cohort. We used country of birth to classify ethnic minority groups, however, self-report is considered the ‘gold standard’ (Kaufman, 1999), given that country of birth captures only a limited aspect of ethnicity and may not align with self-reported ethnic identity or perceived ethnicity. First-generation migrants in our cohort who landed before 1985 or who landed outside of Ontario, second-generation or higher migrants, refugee claimants awaiting the decision on their status and temporary non-status immigrants will be misclassified in the general population reference group, suggesting that we may have underestimated the effects of ethnicity on the risk of involuntary admission. Although we examined ethnic minority groups in more detail than previous studies, the classification system we used is still broad, and there remains a great deal of heterogeneity within each group. We limited follow-up to 2 years after diagnosis to capture involuntary admissions occurring early in the course of illness; however, this is a relatively short follow-up duration and our findings are not generalisable to admissions occurring after 2 years. Our findings are also not generalisable to rural areas due to exclusion of people with a rural place of residence.

## Conclusions

Our findings have contributed important evidence that ethnic disparities in involuntary admission are present early in the course of psychotic illness. We observed that most first-generation ethnic minority groups with early psychosis have a higher risk of involuntary first admission compared with the general population, with a markedly higher risk among migrants from the Caribbean and Africa. We also observed that refugees from some regions were particularly vulnerable. Taken together with previous Canadian evidence similarly revealing inequities in mental health service use in early psychosis (Anderson *et al*., 2015*b*, 2017), our findings highlight the need for policy initiatives aimed at improving pathways to care in first-generation ethnic minority groups with early psychosis – particularly in Caribbean and African communities. Further studies aimed at understanding the mechanisms behind our findings may shed light on how these inequities can be reduced.

## Supplementary material

For supplementary material accompanying this paper visit https://doi.org/10.1017/S2045796019000556. click here to view supplementary material

## Availability of data and materials

The data set from this study is held securely in coded form at ICES and the ICES analyst (LL) and graduate student (RR) had full access to study data. While data sharing agreements prohibit ICES from making the data set publicly available, access may be granted to those who meet pre-specified criteria for confidential access, available at www.ices.on.ca/DAS. The full dataset creation plan is available from the authors upon request, understanding that the programs may rely upon coding templates or macros that are unique to ICES.
