# Supplementary material for: Risk of involuntary admission among first-generation ethnic minority groups with early psychosis: a retrospective cohort study using health administrative data
Source: Epidemiol Psychiatr Sci. 2019 Oct 15;29:e59. doi: 10.1017/S2045796019000556 (PMC8061249; doi:10.1017/S2045796019000556)
Supplement: Supplementary file 1 [file S2045796019000556sup001.docx]

**Table S1.** Description of cohort definition and variables within the administrative databases, and the codes used

| **Variable Name** | **Source of Data** | **Description** | **Variable Name in Database** | **Database Codes** |
| --- | --- | --- | --- | --- |
| **Index Event** | | | | |
| Incident case of non-affective psychosis | OMHRS, DAD, OHIP, NACRS | - Primary discharge diagnosis of schizophrenia, schizoaffective disorder, or psychosis NOS from a psychiatric bed (OMHRS) or medical bed (DAD) between January 1, 2009 and December 31, 2013,* OR - At least two emergency department or physician visits in a 12-month period for schizophrenia, schizoaffective disorder, or psychosis NOS from January 1, 2009 to December 31, 2013* - Prevalent cases with a history of contact with services prior to 2009 with a diagnostic code for schizophrenia, schizoaffective disorder, or psychosis NOS were excluded | DX10CODE1 (DAD) AXIS1_DSM4CODE_DISCH1 (OMHRS) DX10CODE (NACRS) DXCODE (OHIP) | ICD-9 = 295.X, 298.X ICD-10 = F20, F25, F29 DSM-IV = 295.X, 298.X |
| **Exclusions** | | | | |
| Rural Residence | RPDB | Rural place of residence, defined as residing in a community of ≤ 10,000 | RURAL | 1 = Rural flag  0 = Non-Rural |
| Migrant from Oceania/United States | IRCC | Country of birth recorded for first-generation migrants landing in Ontario (from 1985) | FCOB | Oceania (FCOB = 305, 339, 341-343, 399, 801, 822-826, 830-836, 840-846, 899)  North America (FCOB = 461, 511, 512, 531) |
| Affective or organic psychosis | OMHRS, DAD | Main diagnosis at hospitalization responsible for the longest length of stay was affective or organic psychosis | DX10CODE1 (DAD) AXIS1_DSM4CODE_DISCH1 (OMHRS) | ICD-10 = F302, F312, F315, F323, F333, F105, F115, F125, F147, F155, F165, F195  DSM-IV = 291.3, 291.5, 292.11, 292.12, 293.82, 296.04, 296.24, 296.34, 296.44, 296.54, 296.64 |
| **Exposure Variable** | | | | |
| First-generation ethnic minority group | IRCC | Country of birth recorded for first-generation migrants landing in Ontario (from 1985) | FCOB | 0 = General population (Not included in the IRCC database)  1 = European (Western Europe [FCOB = 11-13, 22, 24, 31, 41, 46, 87, 652, 821], Eastern Europe [FCOB = 14-16, 18-20, 26, 33, 42, 51, 55, 56, 59, 83, 88], Northern Europe [FCOB = 1-10, 17, 21, 27, 32, 40, 85], Southern Europe [FCOB = 25, 28, 30, 34-37, 39, 43, 44, 47, 48, 61-64, 70, 81, 82, 84, 86, 89, 90], Europe Other [FCOB = 99])  2 = Caribbean (FCOB = 601, 602, 605, 610, 620-622, 624-633, 650, 651, 653-658, 699)  3 = South Asian (Southern Asia [FCOB = 201, 205, 209, 212, 254, 264], Asia Other [FCOB = 266, 299, 901, 916]  4 = East Asian (Eastern Asia [FCOB = 198, 200, 202-204, 207, 257, 258, 261, 262, 268], Southeast Asia [FCOB = 121, 122, 152, 153, 186])  5 = Latin American (Central America [FCOB = 501, 541-549], South America [FCOB = 703, 709, 711, 721-725, 751-755, 799])  6 = North African & Middle East (Northern Africa [FCOB = 101, 131, 133, 135, 171, 185], West Central Asia and Middle East [FCOB = 45, 49, 50, 52-54, 57, 58, 60, 206, 208, 210, 213, 221, 223-226, 231, 252, 253, 263, 265, 273, 274, 280]  7 = African (Western Africa [FCOB = 160, 164-167, 169, 170, 173, 174, 176, 177, 180, 181, 187, 188, 911], Eastern Africa [FCOB = 111-113, 130, 132, 136, 154, 161, 162, 172, 175, 179, 182, 183, 902-905], Central Africa [FCOB = 501, 541-549], Southern Africa [FCOB = 121, 122, 152, 153, 186], Africa Other [FCOB = 184, 199, 906, 914, 915]) |
| **Outcome Variable** | | | | |
| Hospitalization for a mental health reason | OMHRS  DAD | First hospitalization to a psychiatric or medical hospital bed for a mental health reason within 2-years following the index event, up to January 1, 2016 | DX10CODE1 (DAD) AXIS1_DSM4CODE_DISCH1 (OMHRS) DX10CODE (NACRS) DXCODE (OHIP) | ICD-10 = F10–F51, F53, F55, F59, F60–F69, F91–F99 DSM-IV = All hospitalizations EXCEPT 290, 293, 294, 299, 302, 314–319, 607–787 and V codes |
| Involuntary Admission | DAD, OMHRS | Involuntary status at first hospital admission for a mental health reason within 2 years of diagnosis | ADMMETH (DAD)  A3A (OMHRS) | 1 = Involuntary admission (ADMMETH = C, D, E; A3A = 1, 4) 0 = No involuntary admission |
| **Covariates/Effect Modifiers** | | | | |
| Age at Index Date | RPDB | Age at index date | BDATE | Continuous variable calculated based on index date and birth date |
| Gender | RPDB | Recorded sex | SEX | 1 = Male  2 = Female |
| Income Quintile | RPDB | Neighbourhood-level income quintile | INCQUINT | 1 = Lowest Income Quintile  5 = Highest Income Quintile |
| Index Diagnosis | DAD, OMHRS, OHIP, NACRS | Diagnostic category of index diagnosis | DX10CODE1 (DAD) AXIS1_DSM4CODE_DISCH1 (OMHRS) DXCODE (OHIP) DX10CODE1 (NACRS) | 1 = Schizophrenia & Schizoaffective Disorder (ICD-10 = F20, F25; DSM-IV = 295.X) 2 = Psychosis Not Otherwise Specified (NOS; ICD-10 = F29; DSM-IV = 298.9) |
| Migrant Class | IRCC | Migrant class for first-generation migrants (immigrant or refugee) | CATEG | 0 = Non-Migrants (Not Included in IRCC Database) 1 = Immigrant (All Codes not Listed Below) 2 = Refugee (CATEG = 020–029, 031–034, 037, 047–049, 052–055, 080, 086–089, 094–095, 120–142, 153) |

*The first event to occur within the case accrual window was considered the index event

**Table S2.** RECORD statement – checklist of items.

|  | **Item No.** | **STROBE items** | **Location in manuscript where items are reported** | **RECORD items** | **Location in manuscript where items are reported** |
| --- | --- | --- | --- | --- | --- |
| **Title and abstract** | | | | | |
|  | 1 | (a) Indicate the study’s design with a commonly used term in the title or the abstract (b) Provide in the abstract an informative and balanced summary of what was done and what was found | (a) Study design reported in abstract  (b) Summary reported in abstract | RECORD 1.1: The type of data used should be specified in the title or abstract. When possible, the name of the databases used should be included.  RECORD 1.2: If applicable, the geographic region and timeframe within which the study took place should be reported in the title or abstract.  RECORD 1.3: If linkage between databases was conducted for the study, this should be clearly stated in the title or abstract. | Specified in title and abstract  Geographic region and timeframe noted in abstract  Specified in the abstract |
| **Introduction** | | | | | |
| Background rationale | 2 | Explain the scientific background and rationale for the investigation being reported | Background and rationale reported in the introduction |  |  |
| Objectives | 3 | State specific objectives, including any prespecified hypotheses | Objectives and hypotheses specified in last paragraph of introduction |  |  |
| **Methods** | | | | | |
| Study Design | 4 | Present key elements of study design early in the paper | Last paragraph of the introduction |  |  |
| Setting | 5 | Describe the setting, locations, and relevant dates, including periods of recruitment, exposure, follow-up, and data collection | Methods, paragraphs 1 and 2 |  |  |
| Participants | 6 | *(a) Cohort study* - Give the eligibility criteria, and the sources and methods of selection of participants. Describe methods of follow-up  *Case-control study* - Give the eligibility criteria, and the sources and methods of case ascertainment and control selection. Give the rationale for the choice of cases and controls  *Cross-sectional study* - Give the eligibility criteria, and the sources and methods of selection of participants  *(b) Cohort study* - For matched studies, give matching criteria and number of exposed and unexposed  *Case-control study* - For matched studies, give matching criteria and the number of controls per case | (a) Paragraph 1 and 2 of methods, supplementary table S1.  (b) N/A | RECORD 6.1: The methods of study population selection (such as codes or algorithms used to identify subjects) should be listed in detail. If this is not possible, an explanation should be provided.  RECORD 6.2: Any validation studies of the codes or algorithms used to select the population should be referenced. If validation was conducted for this study and not published elsewhere, detailed methods and results should be provided.  RECORD 6.3: If the study involved linkage of databases, consider use of a flow diagram or other graphical display to demonstrate the data linkage process, including the number of individuals with linked data at each stage. | Paragraph 1 of methods, supplementary table S1.  Reference cited in methods, paragraph 1. |
| Variables | 7 | Clearly define all outcomes, exposures, predictors, potential confounders, and effect modifiers. Give diagnostic criteria, if applicable. | Methods paragraphs 3 to 5, and supplementary table S1. | RECORD 7.1: A complete list of codes and algorithms used to classify exposures, outcomes, confounders, and effect modifiers should be provided. If these cannot be reported, an explanation should be provided. | Supplementary table S1. |
| Data sources/ measurement | 8 | For each variable of interest, give sources of data and details of methods of assessment (measurement).  Describe comparability of assessment methods if there is more than one group | Methods paragraphs 3 to 5, and supplementary table S1. |  |  |
| Bias | 9 | Describe any efforts to address potential sources of bias | Methods paragraph 2, discussion, strengths and limitations section |  |  |
| Study size | 10 | Explain how the study size was arrived at | Methods paragraph 2, results paragraph 1 |  |  |
| Quantitative variables | 11 | Explain how quantitative variables were handled in the analyses. If applicable, describe which groupings were chosen, and why | Methods, data analysis section |  |  |
| Statistical methods | 12 | (a) Describe all statistical methods, including those used to control for confounding  (b) Describe any methods used to examine subgroups and interactions  (c) Explain how missing data were addressed  (d) *Cohort study* - If applicable, explain how loss to follow-up was addressed  *Case-control study* - If applicable, explain how matching of cases and controls was addressed  *Cross-sectional study* - If applicable, describe analytical methods taking account of sampling strategy  (e) Describe any sensitivity analyses | (a) Methods, data analysis section  (b) Methods, data analysis section, paragraph 2  (c) Described in methods, data analysis section, paragraph 1.  (d) N/A  (e) Last paragraph of methods |  |  |
| Data access and cleaning methods |  | .. |  | RECORD 12.1: Authors should describe the extent to which the investigators had access to the database population used to create the study population.  RECORD 12.2: Authors should provide information on the data cleaning methods used in the study. | Acknowledgements  N/A |
| Linkage |  | .. |  | RECORD 12.3: State whether the study included person-level, institutional-level, or other data linkage across two or more databases. The methods of linkage and methods of linkage quality evaluation should be provided. | Methods, paragraph 1 |
| **Results** | | | | | |
| Participants | 13 | (a) Report the numbers of individuals at each stage of the study (*e.g.*, numbers potentially eligible, examined for eligibility, confirmed eligible, included in the study, completing follow-up, and analysed)  (b) Give reasons for non-participation at each stage.  (c) Consider use of a flow diagram | (a) Results, paragraph 1  (b) N/A  (c) Flow diagram in supplementary figure 1 | RECORD 13.1: Describe in detail the selection of the persons included in the study (*i.e.,* study population selection) including filtering based on data quality, data availability and linkage. The selection of included persons can be described in the text and/or by means of the study flow diagram. | Results, paragraph 1, supplementary figure 1 |
| Descriptive data | 14 | (a) Give characteristics of study participants (*e.g.*, demographic, clinical, social) and information on exposures and potential confounders  (b) Indicate the number of participants with missing data for each variable of interest  (c) *Cohort study* - summarise follow-up time (*e.g.*, average and total amount) | (a) Results, paragraph 1, Tables 1  (b) Described in methods. Missing data in other variables was negligible (< 1%)  (c) Results, paragraph 2 |  |  |
| Outcome data | 15 | *Cohort study* - Report numbers of outcome events or summary measures over time  *Case-control study* - Report numbers in each exposure category, or summary measures of exposure  *Cross-sectional study* - Report numbers of outcome events or summary measures | Table 2 |  |  |
| Main results | 16 | (a) Give unadjusted estimates and, if applicable, confounder-adjusted estimates and their precision (e.g., 95% confidence interval). Make clear which confounders were adjusted for and why they were included  (b) Report category boundaries when continuous variables were categorized  (c) If relevant, consider translating estimates of relative risk into absolute risk for a meaningful time period | (a) Table 2  (b) N/A  (c) N/A |  |  |
| Other analyses | 17 | Report other analyses done—e.g., analyses of subgroups and interactions, and sensitivity analyses | Results paragraph 2, 3, 4, Tables 3 & 4 |  |  |
| **Discussion** | | | | | |
| Key results | 18 | Summarise key results with reference to study objectives | Paragraph 1 |  |  |
| Limitations | 19 | Discuss limitations of the study, taking into account sources of potential bias or imprecision. Discuss both direction and magnitude of any potential bias | Discussion, strengths and limitations section | RECORD 19.1: Discuss the implications of using data that were not created or collected to answer the specific research question(s). Include discussion of misclassification bias, unmeasured confounding, missing data, and changing eligibility over time, as they pertain to the study being reported. | Discussion, strengths and limitations section |
| Interpretation | 20 | Give a cautious overall interpretation of results considering objectives, limitations, multiplicity of analyses, results from similar studies, and other relevant evidence | Discussion |  |  |
| Generalisability | 21 | Discuss the generalisability (external validity) of the study results | Discussion, strengths and limitations section |  |  |
| **Other Information** | | | | | |
| Funding | 22 | Give the source of funding and the role of the funders for the present study and, if applicable, for the original study on which the present article is based | Acknowledgments |  |  |
| Accessibility of protocol, raw data, and programming code |  | .. |  | RECORD 22.1: Authors should provide information on how to access any supplemental information such as the study protocol, raw data, or programming code. | Acknowledgments |

*Reference: Benchimol EI, Smeeth L, Guttmann A, Harron K, Moher D, Petersen I, Sørensen HT, von Elm E, Langan SM, the RECORD Working Committee. The REporting of studies Conducted using Observational Routinely-collected health Data (RECORD) Statement. *PLoS Medicine* 2015; in press.

*Checklist is protected under Creative Commons Attribution ([CC BY](http://creativecommons.org/licenses/by/4.0/)) license.

**
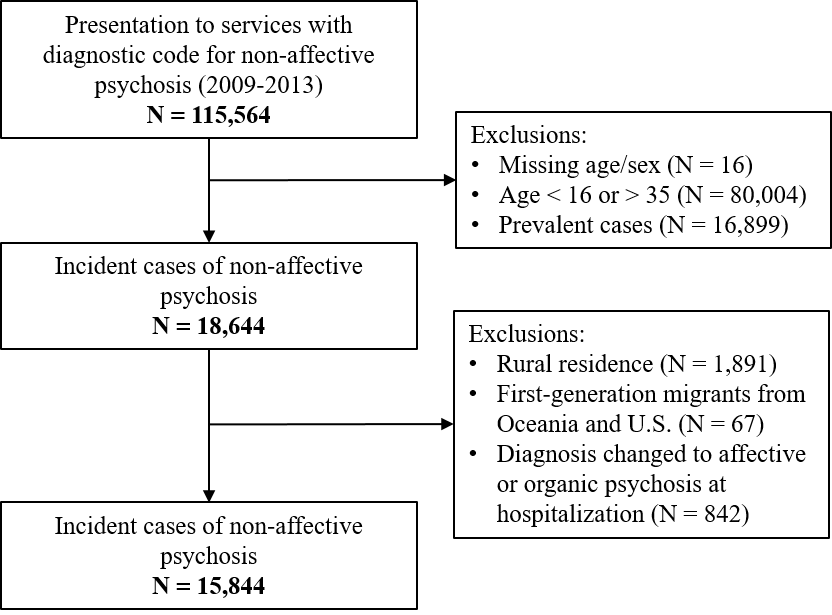
**

**Figure S1.** Flow diagram of cohort inclusion and exclusion numbers.

**Table S3.** Risk of a first hospitalization among first-generation ethnic minority groups within two years of psychosis onset.

| **Ethnic minority group** | **N** | **N (%)** | **Unadjusted RR**  **(95% CI)** | **Adjusted RR^a^**  **(95% CI)** |
| --- | --- | --- | --- | --- |
| General population | 12,795 | 4,445 (35) | Reference | Reference |
| European | 547 | 197 (36) | 1.04 (0.92, 1.16) | 1.05 (0.94, 1.17) |
| African | 405 | 187 (46) | 1.33 (1.19, 1.48) | 1.36 (1.22, 1.51) |
| Caribbean | 303 | 143 (47) | 1.36 (1.20, 1.53) | 1.37 (1.21, 1.55) |
| South Asian | 652 | 211 (32) | 0.93 (0.83, 1.04) | 1.01 (0.90, 1.13) |
| East Asian | 450 | 172 (38) | 1.10 (0.98, 1.24) | 1.15 (1.02, 1.29) |
| Latin American | 227 | 82 (36) | 1.04 (0.87, 1.24) | 1.09 (0.92, 1.30) |
| North African & Middle East | 465 | 173 (37) | 1.07 (0.95, 1.21) | 1.12 (0.99, 1.27) |

RR = risk ratio, CI = confidence interval

^a^Adjusted for age, gender, income quintile, and diagnosis (schizophrenia spectrum disorder versus psychosis NOS)
